# Global Food Security and Sustainability

**DOI:** 10.3390/foods14111983

**Published:** 2025-06-04

**Authors:** Theodoros Varzakas, Costas Stathopoulos

**Affiliations:** 1 Department Food Science and Technology, University of the Peloponnese, Antikalamos, 24100 Kalamata, Greece; 2 Food Futures Institute, School of Medical Molecular and Forensic Sciences, Murdoch University, Food Innovation Precinct WA, Room G.05, 37 Dollyup Street, Perth, WA 6181, Australia; costas.stathopoulos@murdoch.edu.au

## 1. Main Text

This Editorial introduces the Special Issue “Editorial Board Members’ Collection Series: “Global Food Security and Sustainability””, and highlights key topics on this subject. Sixteen papers have been published, amongst which ten were original research and six were reviews.

They are all within the wide area of food security/insecurity and sustainability.

In the first contribution by Xiao et al. [Contribution 1], the spatial and temporal patterns of the rate of grain self-sufficiency (RSSG) at the county level in Guangdong Province from 2014 to 2023 were reported, proposing policy recommendations such as the prioritization of farmland preservation; the implementation of the “storing grain in the land” strategy to ensure food supply; the adoption of advanced agricultural technologies for the improvement of grain yield; the reduction in grain loss by the improvement of storage management and the enhancement of storage efficiency; and the facilitation of interdepartmental coordination.

The second contribution refers to the transition toward sustainable food, which requires integrated policies and collaborative action. Key commodity/supply chain groups need to be established. “Net-Map Analysis of the Italian Wheat Supply Chain” is well evidenced, analyzing the different stakeholder relationships and their complexities.

Sustainable food supply chains need to be taken into account as discussed by Dania et al. [1], depicting both vertical and horizontal collaboration approaches.

The third contribution by Ayuso et al. [Contribution 3] deals with dietary fiber (DF) and, more specifically, soluble dietary fiber, a key characteristic of nutrition that affects food security and sustainability. DF is considered an essential component of a healthy diet, and has been associated with several health benefits, reducing mortality from diseases. The authors reported that higher-value-added ingredients could arise from the revalorization of carob, artichoke, apple, and broccoli byproducts after enzymatic treatment, thus improving their nutritional and physicochemical properties.

The fourth contribution by Mastos et al. [Contribution 4] investigated the performance of sustainable supply chain management (SSCM) in the Greek food industry by using structural equation modeling. They concluded that society and the environment are affected by supply chain strategic orientation, and that they both affect economics. Moreover, firm-level critical sustainability factors play a determining role.

The fifth contribution by Loi et al. [Contribution 5] addressed the issue of mycotoxins as a key factor in global food security through the Horizon 2020 project. The authors referred to risk assessment and management strategies through the adoption of the harmonization of methods and marketable solutions.

The sixth contribution by Lima et al. [Contribution 6] deals with nanoemulsions as a means of food security in order to enhance essential oils’ dispersion and protection. Natural preservatives instead of synthetic ones are a key priority in food systems.

Consumers show preference for low-greenhouse gas (GHG)-emission produce [2], as well as the declaration of a carbon footprint (e.g., the European Commission’s Product Environmental Footprint). Hence, in the seventh contribution by Simmons et al. [Contribution 7], strategies for reducing the carbon footprint of cherry and orange production were examined, including the avoidance of emissions by sequestering C from the atmosphere.

The eighth contribution by Lee et al. [Contribution 8] considered global food safety systems and standards, addressing many case studies and issues related to food safety culture in developed and developing countries.

In the ninth contribution by Ajayi et al. [Contribution 9], the issue of bioactive peptides from plant-based proteins was explored, which has been gaining attention due to their promotion of human health and alleviation of diseases. In this direction, quinoa protein hydrolysates (QPHs) have been considered as an alternative therapy for the treatment of hypercholesterolemia.

The tenth contribution by Kotsou et al. [Contribution 10] dealt with edible insects as well as *Tenebrio molitor*, and their enhancement through the addition of albedo orange peel waste.

The eleventh contribution by Varzakas and Antoniadou [Contribution 11] dealt with food ethics and ethically informed food choices. The spiritual and physical significance of food were determined through the perspective of oral health aiding holistic aspects of well-being. Ethical practices and transparency will work their way towards more sustainable and equitable food systems.

The twelfth contribution by Varzakas and Smaoui [Contribution 12] brought to the forefront the whole dimension of sustainable food systems (SFSs), as connected to the Sustainable Development Goals (SDGs). A plant-based protein case study was well evidenced.

The thirteenth contribution examined food products developed using *T. molitor* larvae, addressing issues of food security and environmental sustainability linked to nutrition.

The fourteenth contribution by Vinas-Ospino et al. [Contribution 14] addressed the issue of green solvent technology offering significant advantages in terms of extracting carotenoids from fruit and vegetable byproducts. This is a major aspect of food security and sustainability.

The fifteenth contribution by Carvalho et al. [Contribution 15] employed a holistic way of considering sustainability, uniting healthy eating, personal health, and well-being.

Finally, Lara et al. [Contribution 16] described issues of food biodiversity and underutilized and neglected food crops, leading to food and nutrition insecurity. Indigenous crops, landrace varieties, and diversification are some of the proposed strategies underlined.

## 2. Conclusions

Global food security and sustainability issues need to be addressed in a holistic way, taking into account all stakeholders’ needs and expectations. All of the issues raised above will aid in safeguarding our health and the health of our planet; this being the case, the mission is to achieve this, and this will involve a coordinated effort from all of us. It is in our hands.

## Data Availability

Not applicable.
